# Functional study of the voice and swallowing following supracricoid laryngectomy

**DOI:** 10.1016/S1808-8694(15)31059-4

**Published:** 2015-10-22

**Authors:** Nair Katia Nemr, Marcos Brasilino de Carvalho, Juliana Köhle, Grazielle Capatto de Almeida Leite, Abrão Rapoport, Regis M. Scheffer Szeliga

**Affiliations:** 1 Doctor in Social Psychology, graduated at USP. Teacher and Speech Therapist.; 2 Doctor in Medicine, trained at UNICAMP, Head & neck surgeon. Teacher in the Health Sciences post-graduate course at the Heliopolis Hospital.; 3 Voice specialist, speech therapist.; 4 Master's degree in speech therapy, trained at PUC-SP. Voice specialist. Speech therapist at the Speech Therapy Unit of the Heliopolis Hospital.; 5 Teacher at the University of Sao Paulo. Head & neck surgeon. Coordinator of the do Health Sciences post-graduate course at the Heliopolis Hospital.; 6 Head & neck surgery specialist certified by the Brazilian Head & Neck Surgery Society and the Brazilian Medical Association. Head & neck surgeon.; 7 Heliopolis Hospital, Sao Paulo, SP, Brazil.

**Keywords:** swallowing, supracricoid laryngectomy, voice

## Summary

**T**o identify the grade and evolution of dysphagia and dysphonia in patients undergoing supracricoid laringectomy, and to study the association of these findings with clinical and surgical variables. **Method:** The study included 22 cases undergoing supracricoid laringectomy at the Head and Neck Surgery and Otolaryngology Department of the Heliopolis Hospital - Brasil, and referred to speech therapy. Dysphagia and dysphonia were correlated with gender, age, stage T (T1, T2, T3, T4), primary site (supraglottis, glottis or subglottis), preservation of one or two arytenoids, reconstructive procedures (cricohyoidopexy or cricohyoido epiglotopexy), time to withdraw the naso-enteral tube, and time to close the tracheostomy. Statistical tests included the Chi-square and/or Fischer's exact test. **Results:** We observed an association between moderate grade dysphagia and the glottis as the primary site, cricohyoidoepiglotopexy as the type of reconstruction and naso-enteral tube removal within one month after the surgery. There was also an association between severe dysphagia and the supraglottis as the primary site. Dysphagia and dysphonia were associated in the degree of severity; however a larger number of patients had better progression of dysphagia compared to the progression of dysphonia. There was no statistical significance between other associations. **Conclusion:** Improvement of swallowing is more frequent than improvement of dysphagia. There is an association between moderate dysphagia and the glotttis as primary site, cricohyoidoepiglotopexy and naso-enteral tube removal within one month after surgery.

## INTRODUCTION

The percentage of laryngeal cancer worldwide is 1 to 2% of malignant tumors and is associated with smoking, alcohol abuse, professional exposure to chemical agents and a family history of cancer. Generally it affects men between 50 and 60 years, although women and individuals of any age may also develop this type of cancer.1 The surgical procedure usually recommended for this malignancy is partial, subtotal or total laryngectomy.

Supracricoid laryngectomy, which is a form of subtotal laryngectomy, was initially described by Majer and Rieder in 1959^2^ and thereafter by Labayle and Bismuth in 1972.^3^ This procedure was developed to avoid total laryngectomy in patients with tumor in which traditional partial procedures would not be indicated, thus avoiding definitive tracheostomy and the irreversible loss of laryngeal voice. In supracricoid laryngectomy for transglottic tumors with glottic and supraglottic involvement and minimal extension to the infraglottis, most of the larynx is removed. The hyoid bone, the cricoid cartilage, and at least one arytenoid are preserved, thus maintaining the possibility of functional reconstruction. There are two forms of laryngeal reconstruction: cricohyoidopexy (CHP), in which the cricoid cartilage is placed closer to the hyoid bone (for supraglottic tumors non-resectable by supraglottic laryngectomy), and cricohyoidoepiglottopexy (CHEP), in which the epiglottis is maintained and its lower portion is included in the suture that approximates the cricoid to the hyoid bone (for glottic region tumors).^4^,^5^ Function is generally superior when the epiglottis is preserved. The main advantage of this type of resection is the possibility of a temporary tracheostomy with voice preservation, albeit hoarse and soprous.

The functional aspects of these surgical procedures have not been widely described in medical literature. Speech therapy following surgery has increased the possibility of adequate swallowing, normal feeding (after removal of the nasoenteric tube and closure of the traqueostomy), and improved voice.

This paper aims to identify the grade and progression of dysphagia and dysphonia by speech therapy assessments in patients undergoing supracricoid laryngectomies; a second aim it to investigate the association between the grade and progression of dysphagia and dysphonia and functional progression with gender, age, tumor site, T staging, preservation of one or two arytenoids, type of reconstruction (CHP or CHEP), time from removal of the nasoenteric tube, and closure of the tracheostomy.

## METHODS

The series included 22 individuals who had undergone supracricoid laryngectomy and then been referred to the Phonoaudiology Unit of the Heliopolis Hospital in Sao Paulo, Brazil, between 1987 and 2003.

Swallowing was assessed clinically by using criteria adapted from O'Neil et al.'s^6^ protocol: 1- adapted swallowing (normal swallowing for all food consistencies, or difficulty during the oral or pharyngeal phases with spontaneous compensation and cleaning of residual food and no signs of tracheal aspiration and/or laryngeal penetration); 2- mild dysphagia (slight aspiration of liquids, with a cough reflex); 3- moderate dysphagia (aspiration for two or more food consistencies, with or with no cough reflex and good compensation during postural maneuvers); 4- severe dysphagia (aspiration for two or more food consistencies, with or with no cough reflex and no compensation during maneuvers, and an indication for enteral feeding).

Voice was analyzed according to Pinho's classification,7,8 which is based on an auditory-perceptive evaluation: 0- normal voice (absence of hoarseness, roughness, soprosity, asthenia or tension); 1- mild dysphonia (hoarse/rough/tense voice with sonorization); 2- moderate dysphonia (hoarse/rough/tense/soprous voice with non-systematic sonorization); 3- severe dysphonia (voice with no sonorization). These criteria were established independently from the grade of hoarseness, roughness, soprosity, asthenia, and tension.

We considered the progression of dysphagia and dysphonia as follows: satisfactory (when there was improvement observed by changes in the functional classification scale during speech therapy), or unsatisfactory (when dysphagia or dysphonia did not progress from its present grades, or when there was worsening in the classification scale, such as in cases of completion laryngectomy, regardless of cause).

General functional outcome was classified as: satisfactory (improvement both in the grade of dysphagia and dysphonia), or unsatisfactory (when there was no improvement in one or both functions).

The Chi-squared test (X2) and/or Fisher's exact test were used for statistical analysis; the significance level was considered for values lower than or equal to 5% (p ≤ 0.05).

The study project was approved by the Research Ethics Committee of the Heliopolis Hospital, with the number 280.

## RESULTS

Thirteen patients (59%) presented moderate grade dysphagia, and 9 patients (41%) had severe dysphagia. Nine patients (41%) presented moderate grade dysphonia, and 13 patients (59%) had severe dysphonia.

Table 1 shows the association between the grade of dysphagia and gender, age, tumor site, T staging, preservation of the arytenoids, reconstruction, nasoenteric tube, tracheostomy, progression of dysphagia, and general outcome.

**Table 1:** Correlation between the grade of dysphagia and gender, age, site, staging, arytenoid, reconstruction, nasoenteric tube, tracheotomy, progression of dysphagia and general outcome.

		Gender		Age		Tumor site		Staging		Arytenoid		Reconstruction		nasoenteric tube		Tracheotomy		Progression of Dysphagia		General outcome	
		M	F	> 50	≤ 50	Glottis	Supra Glottis	T1T2	T3	1	2	CHP	CHEP	< 1 month	> 1 month	< 1 month	> 1 month	Satisf.	Unsatisf.	Satisf.	Unsatisf.
Grade of Dysphagia	Mod.	10 (59%)	3 (60%)	10 (63%)	3 (50%)	11 (85%)	2 (22%)	8 (67%)	5 (50%)	9 (52%)	4 (80%)	1 (14%)	12 (80%)	8 (89%)	5 (45%)	5 (100%)	6 (50%)	13 (65%)	0	10 (67%)	3 (43%)
	Sev.	7 (41%)	2 (40%)	6 (37%)	3 (50%)	2 (15%)	7 (78%)	4 (33%)	5 (50%)	8 (48%)	1 (20%)	6 (86%)	3 (20%)	1 (11%)	6 (55%)	0	6 (50%)	7 (35%)	2 (100%)	5 (33%)	4 (57%)
Total		17 (100%)	5 (100%)	16 (100%)	6 (100%)	13 (100%)	9 (100%)	12 (100%)	10 (100%)	17 (100%)	5 (100%)	7 (100%)	15 (100%)	9 (100%)	11 (100%)	5 (100%)	12 (100%)	20 (100%)	2 (100%)	15 (100%)	7 (100%)
X2		0.96		0.59		0.003 (*)		0.42		0.27		0.003 (*)		não aplicado		não aplicado		0.07		0.07	
Fischer		0.68		0.47		0.005 (*)		0.36		0.29		0.006(*)		0.05(*)		0.07		0.15		0.15	

**Key for**Table 1

Mod.= moderate

Sev. = severe

M = male

F = female

> 50 = over 50 years

≤ 50 = less or equal to 50 tears

1 = preservation of 1 arytenoid

2 = preservation of 2 arytenoids

CHP = cricohyoidopexy

CHEP = cricohyoidoepiglottopexy

< 1 month = up to 1 month postoperative

> 1 month = more than 1 month postoperative

Satisf. = satisfactory

Unsatisf. = unsatisfactory

(*) = significant p value

The association between moderate grade dysphagia, the glottis as the primary site, and CHEP as the reconstruction technique was statistically significant, as was the association between severe dysphagia, the supraglottis as the primary site and CHP.

Statistical analysis of the associations established between the grade and progression of dysphagia and the removal time of the nasoenteric tube excluded two patients who had the nasoenteric tube removed only after completion laryngectomy. There was statistical significance between the grade of dysphagia and the removal time of the nasoenteric tube, showing that in general, the nasoenteric tube could only be removed one month postoperatively in cases with severe dysphagia.

Table 2 shows the association between the grade of dysphonia and gender, age, tumor site, T staging, preservation of the arytenoids, reconstruction, tracheostomy, progression of dysphonia, general outcome, and grade of dysphagia.

**Table 2:** Correlation between the grade of dysphonia and gender, age, site, staging, arytenoid, reconstruction, tracheotomy, progression of dysphonia, general outcome, and grade of dysphagia.

		Gender		Age		Tumor site		Staging		Arytenoid		Reconstruction		Tracheotomy		Progression of dysphonia		General outcome		Grade of Dysphagia	
		M	F	> 50	≤ 50	Glottis	Supra Glottis	T1T2	T3	1	2	CHP	CHEP	< 1 month	> 1 month	Satisf.	Unsatisf.	Satisf.	Unsatisf.	mod.	sev.
Grade of Dysphonia	Mod.	6 (35%)	3 (60%)	7 (44%)	2 (33%)	7 (54%)	2 (22%)	5 (42%)	4 (40%)	7 (41%)	2 (40%)	1 (14%)	8 (53%)	5 (100%)	6 (50%)	7 (47%)	2 (29%)	7 (47%)	2 (29%)	8 (62%)	1 (11%)
	Sev.	11 (65%)	2 (40%)	9 (56%)	4 (67%)	6 (46%)	7 (78%)	7 (58%)	6 (60%)	10 (59%)	3 (60%)	6 (86%)	7 (47%)	0	6 (50%)	8 (53%)	5 (71%)	8 (53%)	5 (71%)	5 (38%)	8 (89%)
Total		17 (100%)	5 (100%)	16 (100%)	6 (100%)	13 (100%)	9 (100%)	12 (100%)	10 (100%)	17 (100%)	5 (100%)	7 (100%)	15 (100%)	5 (100%)	12 (100%)	15 (100%)	7 (100%)	15 (100%)	7 (100%)	13 (100%)	9 (100%)
X2		0.32		0.65		0.13		0.93		0.96		0.08		não aplicado		0.42		0.42		0.018 (*)	
Fischer		0.31		0.52		0.14		0.63		0.68		0.10		0.56		0.37		0.37		0.024 (*)	

**Key for**Table 2

Mod.= moderate

Sev. = severe

M = male

F = female

> 50 = over 50 years

≤ 50 = less or equal to 50 tears

1 = preservation of 1 arytenoid

2 = preservation of 2 arytenoids

CHP = cricohyoidopexy

CHEP = cricohyoidoepiglottopexy

< 1 month = up to 1 month postoperative

> 1 month = more than 1 month postoperative

Satisf. = satisfactory

Unsatisf. = unsatisfactory

There was a statistically significant association between the grade of dysphagia and the grade of dysphonia. Statistical analysis of the associations established between the grade and progression of dysphagia, the grade and progression of dysphonia and closure of the tracheostomy excluded five patients who remained with opened tracheostomies until the end of this trial, two due to neolaryngeal stenosis (one patient died because of unrelated causes and the other patient is undergoing dilatation of the neoglottis), two others due to completion laryngectomy (recurrence), and one due to loss of follow-up.

A similar progression for dysphonia and dysphagia was seen in 77% of cases (15 patients had a satisfactory progression and 2 patients had an unsatisfactory progression). The progression of dysphagia was satisfactory and the progression of dysphonia was unsatisfactory in 23% of cases. Thus, a higher number of patients had a satisfactory progression of dysphagia (p=0.02).

## DISCUSSION

In medical literature, dysphonia is frequently described as severe in CHEP.^9^ In our series, dysphonia was present in 47% of patients undergoing this form of reconstruction. Most of the patients (68%) showed improvement of their voice pattern following speech therapy, as has been published in medical literature.^10^, ^11^, ^12^, ^13^

Dysphagia, particularly for liquids, is reported in most cases of supracricoid laryngectomy; swallowing function may be improved by speech therapy.^14^,^15^ In our study, 59% of patients had moderate dysphagia. Satisfactory progression was seen in 91% of cases; only 9% persisted with severe dysphagia, progressing to completion laryngectomy due to recurrence.

The statistically significant association between moderate dysphagia and the glottis as the primary site, and severe dysphagia with the supraglottis as the primary site, reinforces the significance seen in similar degrees of dysphagia with CHEP and CHP. Although there was no statistically significant difference in the association between these aspects and the general outcome, there was a higher frequency of satisfactory progression, both for the glottis as the site and for CHEP. Less severe dysphagia was seen in cases where the epiglottis was preserved, underlining its role in swallowing as well as the need for further studies on this topic. There are references to the continuation of severe dysphagia in cases of CHEP where there was no functional dynamic compensation of the epiglottis.^16^

There was no statistically significant difference or noteworthy percentage difference between the presence of one or two arytenoids and functional progression of voice and swallowing. Since only one arytenoid was preserved in most cases, similar to other published trials,^17^ further studies with larger series, in which two arytenoids are preserved, would be enlightening. Considering the functional importance of the arytenoids in the new swallowing conditions, it is reasonable to assume that dysphagia may be reduced further by preserving two arytenoids.

The nasoenteric tube removal time described in medical literature varies from 9 to 90 days postoperatively. In some studies there are reports of normalization and/or significant improvements in voice and swallowing up to one year following surgery.^12^,^18^, ^19^, ^20^ In our series, the nasoenteric tube was removed in 9 patients within the first month after surgery; the nasoenteric tube was removed after the first postoperative month in the remaining 11 patients. Nasoenteric tube removal within one month following surgery has been reported in literature.15 Nasoenteric tube permanence is relevant as it affects the quality of life of patients as well as the clinical strategy. Following surgery, a significant portion of these patients is referred to radiotherapy, which should be started up to six weeks postoperatively for best results. However, if the tube cannot be removed before, during or after radiation therapy, reintroduction of oral feeding becomes much more difficult.

There was a convergence between closure of the tracheostomy and the degree of dysphagia and nasoenteric tube removal time. When the nasoenteric tube is removed, closure of the traqueostomy usually follows suit. A similar situation is seen in the association between the degree of dysphonia and the tracheostomy closure time. However, it should be said that not only does an open tracheostomy interfere negatively on voice improvement, but its permanence is cases of supracricoid laryngectomy, except for neolaryngeal stenosis, is due to dysphagia and the risks of tracheal aspiration. Furthermore, an open tracheostomy and the cannula will also interfere with laryngeal elevation during swallowing.21 Time for removal of the cannula was equal to or less than 30 days in 5 cases (29%), and more than 30 days in 12 cases (71%), out of 17 cases. Other studies have suggested that normal breathing will resume after between 18 days and one year.^1^,^8^

As expected, we observed that most patients improved more of dysphagia than dysphonia (swallowing progressed satisfactorily in 23% of cases, but with unsatisfactory voice improvement). Even so, there was a high rate of functional satisfactory progression for both conditions (68%). Medical literature appears to agree that most patients undergoing supracricoid laryngectomy have a satisfactory improvement of function.^4^,^10^,^14^,^22^

Given its restricted indications, supracricoid laryngectomy needs to be further studied to substantiate the best possible improvement in voice and swallowing function for those patients who require this approach. This is a relevant theme for speech therapy clinics that focus on head & neck surgery patients. Integration between both areas is essential when caring for these patients, especially in cases where surgery walks the border between partial and total laryngectomy. Obtaining equivalent oncological results does not preclude the need to reach higher standards of quality of life, which result from more effective functional adaptation.

## CONCLUSION

In patients undergoing supracricoid laryngectomy, improvement in swallowing is more satisfactory than the improvement of dysphonia. With the aid of speech therapy, the outcome of both dysphagia and dysphonia was improved when the primary tumor site was the glottic region and allowed reconstruction with cricohyoidoepiglottopexy.
